# Long-Term Outcome of Surgically Repaired and Non-repaired Double Aortic Arch in Children

**DOI:** 10.7759/cureus.60463

**Published:** 2024-05-16

**Authors:** Sara G Hamad, Mohammed Sawahreh, Abdel Rahman A E’mar, Ahmed Abushahin, Mutasim Abu-Hasan

**Affiliations:** 1 Pediatric Pulmonology, Hamad Medical Corporation, Doha, QAT; 2 Pediatric Medicine, Sidra Medicine, Doha, QAT; 3 Pediatric Pulmonology, Sidra Medicine, Doha, QAT

**Keywords:** double aortic arch (daa), vascular ring, conservative management, surgical outcome, children

## Abstract

Introduction

A double aortic arch (DAA) is a rare congenital vascular anomaly that encircles the trachea and esophagus, resulting in compression of both structures and causing variable symptoms of wheezing, stridor, increased work of breathing, or dysphagia. DAA usually presents in infancy but can be incidentally found later in life. The standard management of DAA is surgical repair. However, observation and follow-up have been recommended in asymptomatic or mild cases. The long-term outcome of surgical repair versus observation is not well-reported. We described the long-term clinical outcome of patients with DAA who were surgically repaired versus non-repaired at our institution.

Methods

Electronic medical records were searched for the patients diagnosed with DAA before the age of 18 years. Data from clinical, radiological, and bronchoscopic findings, pulmonary function test (PFT), and cardiopulmonary exercise testing (CPET) were extracted. A structured phone questionnaire of patients’ parents regarding past and current symptoms was also conducted.

Results

A total of 12 patients (eight males four females) with DAA were identified. Median age was 8.5 (1.5-17) years. The age at diagnosis was 60 (1-192) months. Post diagnosis follow-up period was 20 (2-156) months. Five patients were surgically repaired, and seven patients were not repaired. The median age of surgery was five (1-15) years in repaired patients. The phone questionnaire was completed in only 10 patients (five repaired and five non-repaired). Respiratory symptoms in infancy were reported in all repaired and non-repaired patients and were resolved in all five repaired patients and in four of the five non-repaired patients. One non-repaired patient complained of intermittent dyspnea on exertion. Gastrointestinal symptoms were present in infancy in three repaired and three non-repaired patients and were improved in two repaired and one non-repaired patient. PFT was performed in five patients (one repaired, four non-repaired) and showed normal forced expiratory volume in one second (FEV1), forced vital capacity (FVC), and FEV1/FVC in all patients. Low peak expiratory flow (PEF) was seen in the repaired patient and in three of the non-repaired patients. CPET was conducted in four non-repaired patients and showed maximal oxygen consumption (VO2-max) of 66% predicted (58-88), maximal ventilation (VE-max) of 75% predicted (70-104), and ventilatory reserve of 55% predicted (48-104).

Conclusion

Long-term clinical outcome is favorable in both repaired and non-repaired patients with DAA even though both groups reported respiratory symptoms during infancy. Therefore, clinical observation is a legitimate option in certain DAA patients.

## Introduction

A double aortic arch (DAA) is a rare vascular congenital anomaly [1]. The estimated prevalence of vascular rings is about 1%. DAA is considered the most common of complete vascular rings as it accounts for 46-76% of the complete vascular rings, making congenital DAA the most common vascular malformation in the congenital annulus [1,2]. DAA can also be isolated or in conjunction with other congenital cardiac anomalies in around 12.6% of the identified cases. An association with chromosome 22q11 deletion, trisomy 21, and other syndromes was reported in up to 20% of cases [3].

The embryonic development of DAA is due to the persistence of the fourth branchial arch and the dorsal aorta, leading to a complete vascular ring that encircles both the trachea and the esophagus, causing significant symptoms due to compression of both structures [4].

Symptoms of compression include stridor, wheezing, increased work of breathing, and dysphagia. Most affected patients present with symptoms early in infancy [3]. However, DAA can also be asymptomatic and found incidentally later in life by chest imaging [5] or by echocardiography [6], as described in several case reports [7-9].

There is no current consensus on the indications or the long-term outcomes of observational against surgical management.

In symptomatic patients, the standard management is surgical by ligation and division of the non-dominant arch and the ligamentum arteriosum via posterior thoracotomy [10]. Alternatively, observation and follow-up have been recommended in asymptomatic or mild cases, as described in a few case reports [7-9].

Favorable, yet variable, surgical outcomes were described in the literature. Alsenaidi et al. [11] reported the largest review of surgically repaired 81 patients with DAA. Nonetheless, residual respiratory symptoms have been reported in around half of the patients (54%) post-surgical repair. Postoperative tracheal stenosis (14%) and tracheomalacia (7%) were also documented and attributed to the maldevelopment of the trachea caused by vascular compression [12]. Van Son et al. [13] reported 18 patients with DAA whose symptoms recovered postoperatively after a mean follow-up of around 19 years. Anand et al. [14] reported that 36% of the 14 followed patients with DAA had persistent respiratory symptoms after an interval follow-up of four years. Chun et al. [15] also reported that 47% of the studied 11 patients had only mild residual symptoms.

In this study, we describe the clinical, functional, and radiological characteristics and clinical outcomes of patients with repaired and non-repaired double aortic arch at our institution. Our aim is to provide a clinical review of both repaired and non-repaired cases, which would lead to a better understanding of the optimal management strategies for patients with DAA and improve their outcomes.

## Materials and methods

The study was primarily a retrospective study with a phone questionnaire of past and current reported symptoms. The electronic medical records of all patients who were diagnosed with DAA before the age of 18 years and who were followed at our institution were reviewed between January 2017 and June 2021. This study was conducted at Sidra Medicine, Qatar, which represents the sole pediatrics tertiary hospital that commissioned surgical and pediatric cardiac outpatient clinics in early 2018. The study included patients who were operated on at our institution from 2018 to 2021, in addition to patients who were operated on outside of our institution prior to that period (2011-2017) and continued to follow at our subspecialties clinics.

The data on clinical observations and radiological findings were extracted from medical charts. At our institution, spirometry and cardiopulmonary exercise testing (CPET) were offered for children above six years of age as part of pulmonary function tests (PFTs) to evaluate lung function and exercise capacity, especially since some of the patients in our cohort had exertional symptoms. The spirometric data of the PFT and CPET were also collected. The type of management and clinical outcome were also documented. A structured phone questionnaire was conducted to obtain the past (in infancy) and current symptoms, as reported by the parents of the patients. The questionnaire was performed over the phone due to COVID-19 restrictions at the time of the study. It was in either Arabic or English according to the preferred language of the parents. The parents were informed that their participation was voluntary and that the research was anonymous and confidential. The historical data from the parents who agreed to answer our questionnaire were collected in the study, while the historical data from the parents who declined or were unreachable were not incorporated in the study. The parents/caregivers of the involved patients were called by one of the study team members. The questions and clinical signs, such as tachypnea, wheezing, and stridor, were discussed and explained to the parents/caregivers. Some of the clinical information such as the recurrence of chest infections was verified from medical charts. A copy of the questionnaire is available in Appendix 1.

The parents’ reported respiratory, gastrointestinal, and cardiac symptoms and growth were evaluated as part of the structured questionnaire. The data about the outcomes were extracted as a combination of the reported symptoms by the parents, growth charts, and spirometry/CPET.

The results of the performed radiological studies such as the chest radiographs, chest computed tomography-angiography (CTA), and barium esophagography were collected. Video-fluoroscopic swallowing studies (VFSS) and flexible bronchoscopy results were also collected.

Statistical analysis was limited by the small sample size, which is anticipated in rare diseases and small countries such as Qatar. We reported the results as descriptive statistics, such as frequencies and percentages, and medians with (ranges) and/or standard deviations.

The study was approved by the Institutional Review Board of Sidra Medicine (IRB#1772525) on 27/7/2021.

## Results

A total of 12 patients with DAA were identified, with a male-to-female ratio of 2:1. The median age at assessment was 8.5 (1.5-17) years. The median age at diagnosis was 60 (1-192) months. The interval period between diagnosis and the time of conducting the study (follow-up interval) was at a median of 20 (2-156) months. Three of our patients had a follow-up interval of less than a year. Those three patients were the cases that were discovered incidentally and were not surgically repaired, and their families reported mild or no symptoms for years prior to the diagnosis of DAA.

Nine patients (75%) had right aortic arch dominance. Other associated cardiac lesions were identified in only three patients. The associated cardiac lesions were aortic insufficiency, epicardial cyst, and ventricular septal defect (VSD) with duplication of the superior vena cava. Four patients had associated other congenital conditions, including one patient with chromosome 22q11 deletion. The other congenital anomalies included chromosome 7 microdeletion, hemihypertrophy, and pectus excavatum. The diagnosis was incidental in 25% of our patients, but all patients were symptomatic in infancy.

Five patients were surgically repaired, and seven patients were not repaired. The median age at surgical repair was 5 (1-15) years in the five repaired patients. The demographic and clinical characteristics are summarized in Table 1.

**Table 1:** Demographic and clinical characteristics. DAA: Double aortic arch

	Median (Range)
Age at diagnosis	60 (1-192 months)
Follow-up interval	20 (2-156 months)
Age at surgery (for repaired patients, N=5)	5 (1-15 years)
	Number of patients (N%)
Gender	
Male	8 (67%)
Female	4 (33%)
Type of DAA branch dominance	
Right	9 (75%)
Left	2 (17%)
Balanced	1 (8%)
Associated conditions	
Isolated	8 (67%)
Part of other congenital anomalies	4 (33%)
Associated congenital cardiac anomalies	
Present	3 (25%)
Absent	9 (75%)
Incidental diagnosis	
Yes	3 (25%)
No	9 (75%)
Type of management	
Surgical repair	5 (42%)
No surgical repair	7 (58%)
Total patients	12 (100%)

The phone questionnaire was conducted on only 10 patients (five repaired and five non-repaired). Respiratory symptoms in infancy, mainly wheezing, were the most common reported symptoms in all repaired and non-repaired patients and were improved in all five repaired patients and in four out of five non-repaired patients. One non-repaired patient complained of intermittent dyspnea on exertion, leading to exercise limitation. Gastrointestinal symptoms were present in infancy in three repaired and three non-repaired patients and were improved in two repaired patients and one non-repaired patient. Growth retardation was reported and identified in two patients from the repaired group; one of them persisted despite surgical repair. The growth retardation may be attributed to the severity of the DAA symptoms, including tachypnea, retractions, and stridor, which would lead to increased energy expenditure. Meanwhile, none of the patients in the non-repaired group had growth retardation either in infancy or at follow-up. Questionnaire results are summarized in Table 2.

**Table 2:** Reported symptoms in infancy and at follow-up in surgically repaired and non-repaired patients with double aortic arch.

	Repaired DAA (N= 5)		Non-repaired DAA (N=5)	
Symptoms	In infancy, N (%)	Current/Follow-up, N (%)	In infancy, N (%)	Current/Follow-up, N (%)
Respiratory symptoms	5 (100%)	0 (0%)	5 (100%)	1 (20%)
Wheeze	5 (100%)	0 (0%)	5 (100%)	0 (0%)
Stridor	1 (20%)	0 (0%)	2 (40%)	0 (0%)
Recurrent chest infections	3 (60%)	0 (0%)	2 (40%)	0 (0%)
Tachypnea	3 (60%)	0 (0%)	2 (40%)	0 (0%)
Dyspnea	2 (40%)	0 (0%)	1 (20%)	1 (20%)
Retractions	1 (20%)	0 (0%)	1 (20%)	0 (0%)
Gastrointestinal symptoms	3 (60%)	1 (20%)	3 (60%)	2 (40%)
Difficulty in swallowing	2 (40%)	1 (20%)	3 (60%)	2 (40%)
Choking with feeds	3 (60%)	0 (0%)	0 (0%)	0 (0%)
Increased oral secretions	0 (0%)	0 (0%)	2 (40%)	0 (0%)
Gastroesophageal reflux	1 (20%)	0 (0%)	1 (20%)	1 (20%)
Cardiac symptoms	0 (0%)	1 (20%)	1 (20%)	1 (20%)
Cyanosis	0 (0%)	0 (0%)	1 (20%)	0 (0%)
Limitations to physical activity	0 (0%)	1 (20%)	0 (0%)	1 (20%)
Growth retardation	2 (40%)	1 (20%)	0 (0%)	0 (0%)

PFTs were performed in five patients (one in the repaired group and four in the non-repaired group). They showed normal FEV1, FVC, and FEV1/FVC in all patients. Low peak expiratory flow (PEF) was seen in the repaired patients and in three of the non-repaired patients.

CPET was conducted in four non-repaired patients and showed no evidence of exercise limitation and normal cardiovascular, ventilatory, and gas exchange responses. The median maximal oxygen consumption (VO2-max) was 66% (58-88%) predicted, maximal expired ventilation (VE-max) of 75% (70-104%) predicted, and ventilatory reserve of 55% (48-104%) predicted. The findings of PFT and CPET are summarized in Table 3.

**Table 3:** Pulmonary function test and cardiopulmonary exercise test data. FEV1: Forced expiratory volume in 1 second; FVC: Forced vital capacity, FEF25-75%: Forced mid-expiratory flow; PEF: Peak expiratory flow; VO2-Max: Maximal oxygen consumption; VE-max: Maximal expired ventilation

Spirometry Measurements (N=5)	Median (Range)
FEV1% Predicated	90% (71-107%)
FVC% Predicted	96% (89-110%)
FEV1/FVC	80 (70-85)
FEF25-75% Predicted	78% (50-93%)
PEF% Predicted	52% (43-88%)
Cardiopulmonary exercise test measurements (N=4)	
VO2 Max (% Predicted)	66% (58-88%)
VE max (% Predicted)	75% (70-104%)
Ventilatory reserve (% Predicted)	55% (48-104%)

**Figure 1:**
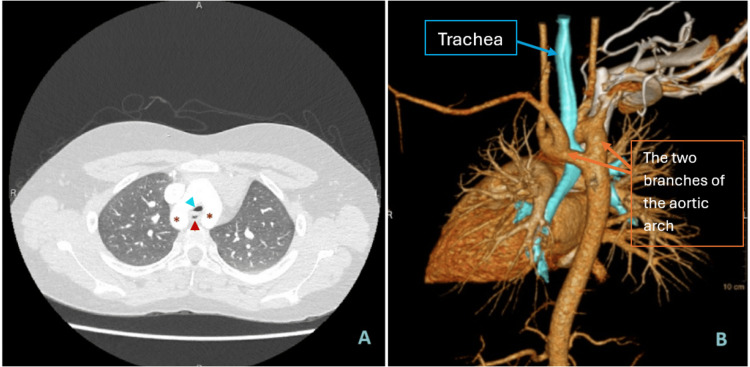
An axial view of computed tomography of the chest in one of the patients enrolled in the study illustrates the balanced double aortic arch (asterisk *) encircling both the trachea (blue arrowhead) and the esophagus (red arrowhead). B: A posterior view of volume-rendered reconstruction of the same patient illustrating the double aortic arch encircling the trachea (esophagus was not re-constructed).

The diagnosis of DAA in our series was established via chest computed tomography-angiography (CTA). A radiological illustration of the anatomical compression is depicted in Figure 1.

Chest radiograph was suggestive prior to CTA in only one patient in our series. Barium esophagography was performed in only five patients and revealed the typical findings of posterior indentation on the esophagus caused by the presence of DAA. VFSS was performed in two patients during infancy, and one of them showed signs of aspiration, while the VFSS was normal in the other patient. Flexible bronchoscopy was performed in five patients in our series and showed pulsatile tracheal compression. One patient had the flexible bronchoscopy prior to the diagnosis by CTA, which suggested and guided the diagnosis of DAA, while the remainder flexible bronchoscopies were performed after establishing the radiological diagnosis of DAA.

## Discussion

In this study, we report the clinical presentation and outcome of surgically repaired and non-repaired cases of DAA at our institution.

DAA is a rare congenital vascular anomaly. Although aberrant right subclavian artery is the most common anomaly arising from the aorta with an incidence of 0.5%-2% worldwide [16], DAA is considered the most common form of complete vascular rings [1,2]. DAA usually manifests in infancy earlier in comparison with other types of vascular rings [13], causing significant respiratory and gastrointestinal symptoms.

There is no specific gender or race inclination. In our study, the male-to-female ratio was 3:1, but we attribute these results to the small number of patients. DAA can be associated with other congenital cardiac anomalies in about 12.6% of the cases. VSD, tetralogy of Fallot, and other complex congenital heart conditions were previously reported [3]. In our cohort, we reported three patients who had an associated congenital cardiac disease, one of them had VSD, while the remaining had insignificant cardiac disease (epicardial cyst and aortic insufficiency).

DAA can also be isolated or in the context of other chromosomal abnormalities, such as chromosome 22q11 deletion and trisomy 21 syndromes. McElhinney et al. studied 66 cases of 22q11 chromosome microdeletions, 14% of which had clinical symptoms of DAA, which became an important etiological factor in DAA [17]. Only one case out of the 12 patients with DAA in our cohort was identified as chromosome 22q11 deletion. The same patient had an isolated finding of DAA with no other associated cardiac anomalies.

Clinical suspicion of a vascular ring, in the context of persistent wheeze and dysphagia, should warrant further investigations to establish diagnosis and to assess for associated congenital cardiac anomalies.

Echocardiography also showed 100% sensitivity in the diagnosis of double aortic arch with both patent branches with similar diameters. Echocardiography showed lower diagnostic accuracy in patients with double aortic arch and atresia of one of its branches [18].

Chest X-rays may be suggestive when the right-sided aortic arch indents the trachea and increases the right paratracheal soft tissue thickness. Contrast swallow studies can also be more helpful. The lateral view demonstrates a posterior indentation caused by the encircling posterior arch [19]. Flexible bronchoscopy may also be suggestive of the diagnosis by finding the typical tracheal compression [20]. The diagnosis is confirmed by CTA, which can also delineate the anatomy for surgical planning [19].

In our study, respiratory symptoms in infancy, mainly wheezing, were the most prevalent symptoms. Life-threatening apneas and cyanosis secondary to reflux have been previously reported [21]. Fortunately, none of the patients in our cohort had a similar presentation.

The reported respiratory symptoms resolved at the time of the study in all interviewed patients, except for one patient who was in the non-repaired group and who reported only intermittent shortness of breath on exertion. Gastrointestinal symptoms resolved in 67% of children in the repaired group, compared with 33% in the non-repaired group. However, the symptoms were not significant or troublesome to patients to necessitate surgical intervention.

In our study, we reported the results of CPET in four patients with DAA. All patients had normal cardiovascular, ventilatory, and gas exchange responses. These results aided the discussion and decision-making of the observational approach. The four patients required no surgical intervention.

To date, there is no available data on CPET in cases of DAA or vascular ring. Our sample size was too small to strongly recommend the utilization of CPAET in the pre-surgical assessment. However, CPET offers an objective tool in the assessment of exercise limitation and outcome measure of the respiratory or cardiac limitation caused by the DAA compression. It may also aid in the decision of surgical repair versus observation approach in older children. However, we understand that this would not be feasible in infants who are the most affected population.

Further prospective and comparative studies are required: To (1) evaluate whether the resolution of symptoms is related to the surgical repair or age-related anatomical changes, (2) develop an individualized approach in children with DAA for surgical indications, and (3) assess time-interval for the resolution of symptoms.

Previous studies have reported variable long-term surgical outcomes. To our knowledge, none described the long-term outcomes in non-repaired cases of DAA. However, the incidental diagnosis of DAA in adulthood and the favorable long-term clinical outcomes in both repaired and non-repaired patients with DAA indicate that clinical observation is a legitimate option in certain DAA patients.

Limitations of the study

Our study reported a small sample size. There is a recall bias given the retrospective design of the study. Additionally, the follow-up period for long-term outcomes is lacking in some patients. The improvement/or persistence of symptoms is difficult to interpret, especially without a detailed analysis of possible correlations with other confounding factors. However, it is the only study that describes both surgically repaired and non-repaired cases of DAA in one institution. We understand that the two groups of patients are different in terms of symptoms and management. Additionally, analyzing and describing these two groups can still provide valuable insights into the outcomes and potential benefits and indications of repair in symptomatic cases. Additionally, our study was the only study to report CPET in children with DAA. We understand that we cannot strongly recommend its use in all cases of DAA. However, CPET utilization in pre-surgical assessments and monitoring may be a target of future studies led by our small group of patients.

## Conclusions

DAA is a rare congenital vascular malformation that can be easily misdiagnosed. Echocardiography combined with CT and airway reconstruction can be diagnosed effectively. Once diagnosis is confirmed, whether the compression on the trachea must exist and whether surgical intervention is required is controversial. However, as for the symptomatic patients, surgical repair is warranted. The airway compression and potential tracheal stenosis caused by DAA is lighter than that of other types of vascular rings, it can be safely observed without surgery, and it is possible to return to normal with its own growth and development. Therefore, clinical observation is a legitimate option in certain DAA patients.

## Appendices

**Table 4:** The questionnaire used for data collection.

Symptoms in Infancy		Current Symptoms (at Follow-up)	
Respiratory Symptoms	Yes/No/Not sure	Respiratory Symptoms	Yes/No/Not sure
Wheeze (Any whistling sound while breathing in infancy – Sound explained)?		Wheeze (Any whistling sound while breathing currently)?	
Stridor (Any noisy breathing in the form of stridor in infancy – Noise explained)?		Stridor (Any noisy breathing in the form of stridor currently)?	
Recurrent chest infections (Any recurrent chest infections (>3 episodes) requiring antibiotics in infancy?)		Recurrent chest infections (Any recurrent chest infections requiring antibiotics in the past year?)	
Tachypnea (Any fast breathing in infancy – Expected respiratory rate (RR) in infancy was explained?)		Tachypnea (Any fast breathing currently– Expected RR for the specific age group was explained?)	
Dyspnea (Any shortness of breath in infancy?)		Dyspnea (Any shortness of breath currently?)	
Chest retractions (Any persistent chest retraction in infancy - Retractions explained)?		Chest retractions (Any persistent chest retraction currently?)	
Gastrointestinal Symptoms	Yes/No/Not sure	Gastrointestinal Symptoms	Yes/No/Not sure
Difficulty Swallowing (Any difficulty in swallowing or pooling of food/milk in infancy)?		Difficulty Swallowing (Any current difficulty in swallowing with solid food or liquids)?	
Choking with Feeds (Any choking with food/milk in infancy)?		Choking with Feeds (Any current choking with solid food or liquids)?	
Increased Oral Secretions (Any excessive drooling of saliva in infancy)?		Increased Oral Secretions (Any current excessive oral secretions or drooling of saliva)?	
Gastroesophageal Reflux (GERD) (Any persistent vomiting, arching of the back, or diagnosis of GERD in infancy)?		Gastroesophageal Reflux (GERD) (Any current persistent vomiting, heartburn, or diagnosis of GERD)?	
Cardiac Symptoms	Yes/No/Not sure	Cardiac Symptoms	Yes/No/Not sure
Cyanosis (Any bluish discoloration around the mouth during infancy?)		Cyanosis (Any current bluish discoloration around the mouth at rest or activity?)	
Limitations in Physical Activity (Any pausing/sweating with feeds or activity in infancy?)		Limitation in Physical Activity (Any current exercise limitation?)	
Growth Retardation	Yes/No/Not sure	Growth Retardation	Yes/No/Not sure
(Any concerns regarding failure to gain weight or grow in infancy?)		Any current concerns regarding growth or failure to gain weight?	
